# Supplementary figures and images for: Malian field isolates provide insight into Plasmodium malariae intra-erythrocytic development and invasion
Source: PLoS Negl Trop Dis. 2025 Jan 6;19(1):e0012790. doi: 10.1371/journal.pntd.0012790 (PMC11735006; doi:10.1371/journal.pntd.0012790)

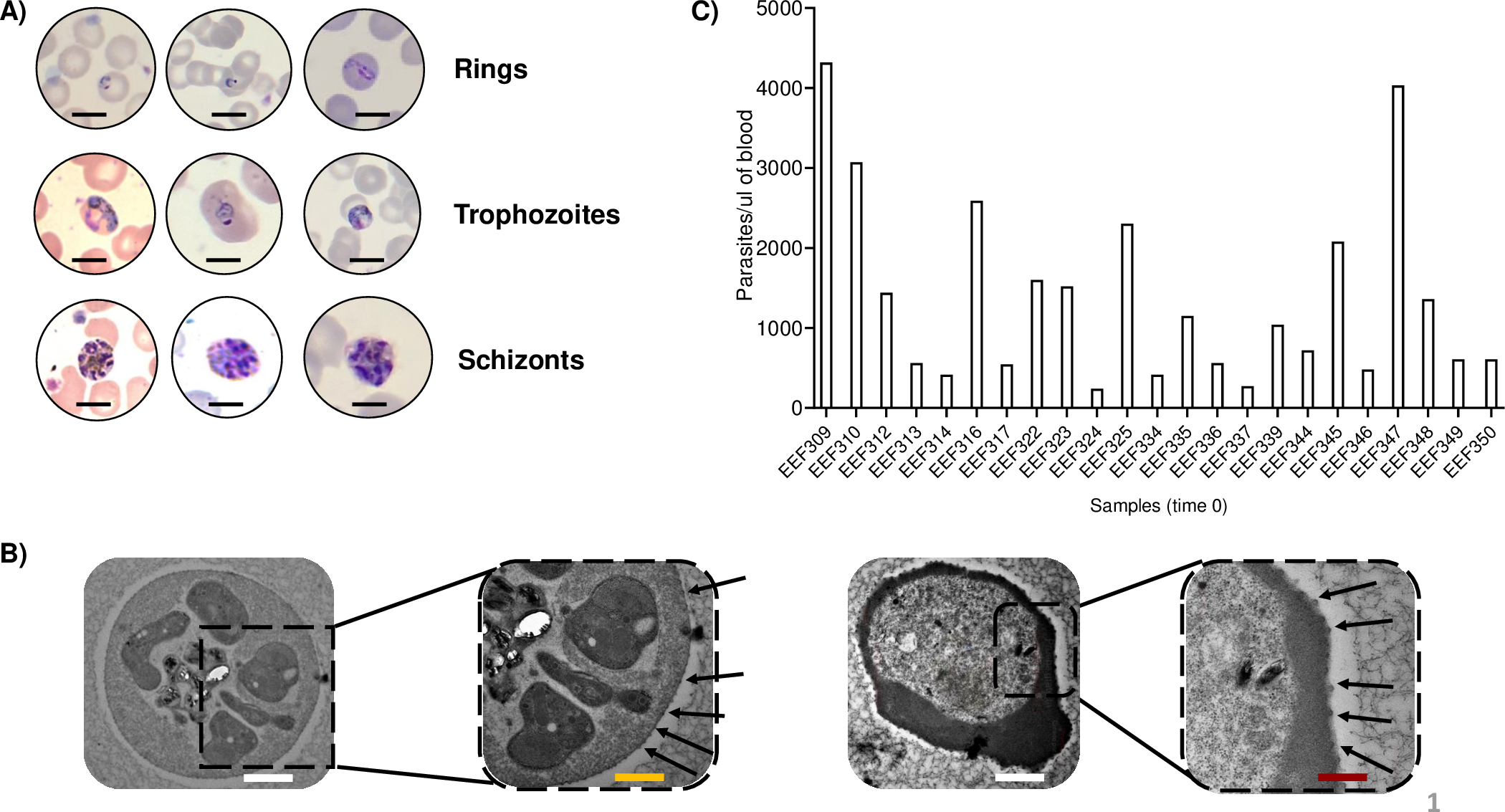

Supplement: S1 Fig — (TIF) [file pntd.0012790.s002.tif]
