# Supplementary material for: Malian field isolates provide insight into Plasmodium malariae intra-erythrocytic development and invasion
Source: PLoS Negl Trop Dis. 2025 Jan 6;19(1):e0012790. doi: 10.1371/journal.pntd.0012790 (PMC11735006; doi:10.1371/journal.pntd.0012790)
Supplement: Attachment — Submitted filename: 241112_Dao_et_al_PLosNTD_Rebuttal_Letter.docx [file pntd.0012790.s005.docx]

**Rebuttal letter**

Dear Editor

Please find the revised version of our manuscript submission (PNTD-D-24-00247R2) - [EMID:f4e62f3a66f2e053] entitled “Malian field isolates provide insight into *Plasmodium malariae* intra-erythrocytic invasion and development”.

Thank you very much again for your review and constructive comments. We have thoroughly examined the manuscript based on the reviewer #4’s comments below. We have addressed all the points/comments made by that reviewer and our point-by-point responses have been included with this letter. A couple of points might deserve your editorial opinion

Please note that all line numbers are referred to the ‘clean’ version of the article, with all changes accepted.

At this third review point, and fourth submission of the paper to your journal, we do believe we have addressed all first three reviewers’ comments and we have not been shy in highlighting the limitations of the study, detailed in the discussion section (line 453-459). Please find below our responses to the new reviewer#4’s comments.

We do look forward to hearing positive news from your side to release this story to your journal readers and the scientific community.

**Reviewer's Responses to Questions**

**Methods**

**Reviewer #1:** no additional comments

**Reviewer #4:**

The objectives of the study are clearly outlined and the methodology employed adequate, as are the materials used. The sample sizes are adequate to address some but not all of the hypothesis tested. There are no specific concerns for the statistical analyses and none for the ethical or regulatory requirements.

Overall, some crucial aspects of the methodology are poorly described or not provided. These must be included in any revised version (see detailed comments below)

**Introduction**

**Line 82: *P. vivax* encodes 11 members of the RBP gene family not only 2.**
Thanks for pointing this out. It has been corrected in the text (line 82).

**Line 97: Reference for CD98 as *P. vivax* reticulocyte receptor is missing (Malleret B, et al.. *Plasmodium vivax* binds host CD98hc (SLC3A2) to enter immature red blood cells. Nat Microbiol. 2021 Aug;6(8):991-999.)**

Thanks for the suggestion to reinforce the introduction. The appropriated reference has been added in the text (line 96).

**Line 112: the review (reference #44) does not mentioned that “*P. berghei* invade reticulocytes 150 times more than normocytes”. The following reference mentioned that PbA was 5.2 times more likely to invade a reticulocyte than a normocyte (Leong YW, et al.. Rodent Malaria Erythrocyte Preference Assessment by an Ex Vivo Tropism Assay. Front Cell Infect Microbiol. 2021 Jul 12;11:680136.)**
Thanks for the comment. Our statement comes from an earlier reference Cromer et al., 2006 (Preferential invasion of reticulocytes during late-stage *Plasmodium berghei* infection accounts for reduced circulating reticulocyte levels). As this is an additional constructive and useful reference and suggestion, we have also incorporated that reference you have suggested reference (Leong YW, et al..2021) (line 111) and changed the amended to:

- ‘*P*. *berghei* can invade reticulocytes with higher preference than normocytes’.

**Line 119: Add references for Flow cytometry and parasitemia/cell tropism monitoring.**

Thank you for the point. A reference has been added (line 118) (Malleret et al., 2011. A rapid and robust tri-color flow cytometry assay for monitoring malaria parasite development)

**It will be useful to describe briefly the culture medium used for *P. malariae* *in vitro* culture, including the type of serum, antibiotics…**
Thank you for the suggestion. You will find a brief description of the culture medium used in the manuscript from line 148 to 151 and a reference for the full culture condition we have published earlier (<https://pubs.acs.org/doi/10.1021/acsinfecdis.1c00262>).

**Line 191: What is the average yield of *P. malariae* after magnetic sorting?**
In the preparatory phase of this study the yield after magnetic sorting was evaluated and showed similar results to previously reported (Ribaut, C., Berry, A., Chevalley, S. *et al.* Concentration and purification by magnetic separation of the erythrocytic stages of all human *Plasmodium* species. *Malar J* **7**, 45 (2008). https://doi.org/10.1186/1475-2875-7-45).

**Line 215: Effect of antibodies in invasion. The authors must use Fab’ fragment antibody to avoid steric hindrance of the antibodies during the *P. malariae* invasion inhibition assay. The antibodies must also be azide-free.**
Thanks for the comment. The antibodies were selected by their known ability to bind the corresponding RBC receptor and the sodium azide was initially never higher than 0.05%. Given the dilutions used in the assays, the final concentration was reduced to a final concentration of 0.005%, which we know is negligible as included in both control and test conditions.

**Line 242: What is the source of immature reticulocyte for the reticulocyte invasion assay? Do the authors enriched the reticulocytes from cord or peripheral blood? If the authors did not add any reticulocytes, it is not an reticulocyte invasion assay but simply a flow cytometry phenotyping to identify CD71 positive Pm-infected red blood cells.**

Thanks for the comment. Reticulocytes were identified from peripheral blood using a conjugated anti-CD71 antibody. According to previous reports ‘*nowadays RNA loss and, above all, the surface expression of CD71 are the most common markers used to define the maturation from reticulocytes to fully mature RBCs’* (Thomson-Luque et al. In-depth phenotypic characterization of reticulocyte maturation using mass cytometry. Blood Cells Mol Dis. 2018 Sep; 72:22–33. doi: [10.1016/j.bcmd.2018.06.004](https://doi.org/10.1016/j.bcmd.2018.06.004)) and previously by Malleret et al. (Significant Biochemical, Biophysical and Metabolic Diversity in Circulating Human Cord Blood Reticulocytes. <https://doi.org/10.1371/journal.pone.0076062>). Therefore, we have used the generally accepted nomenclature for reticulocytes as CD71+ cells, as we are not aware of *P. malariae* being able to invade any other CD71+ cell found in circulation than those from the erythrocyte linage.

Accordingly, we have modified in the material and methods section (k. line 245) the name of the assay to ‘*Identification of P. malariae infected reticulocytes*’*.*

--------------------

**Results**

**Reviewer #1: Figure 4B:** **I would suggest showing the control rather than normalizing to it, so that we can visualize how much the enzymes decreased invasion.**
Thank you for the suggestion, although we have decided to leave the figure with the normalized values as, in our humble opinion, it facilitates interpretation.

**The edits to Figure 6 and its placement within the discussion are much improved and feel more consistent with the findings of this study.**

Thank you for your constructive contributions to this improvement.

**Reviewer #4: There are some concerns with respect to the quality of some of the data/images, as noted below**

**Lines 281-290: It is not clear from the rather image provided that the authors have observed merozoites. From the Giemsa stained picture, as well as from the EM picture, I can only see nuclear masses, NOT fully formed merozoites. The typical mature schizont is characteristically in the form of a central large pigment with the merozoites arranged radially around it. Have the authors seen any of these forms? Whereas the authors have observed some protrusions on the red cell surface, they are suggestive of some membrane “decoration” but not of knobs (as is understood for *P. falciparum*).  Thus in here and in the Discussion, the word “putative” should be added before the word “knob”.**

Based on the literature knowledge, a schizont is multinucleated and segmented form containing daughter merozoites. Please, see more images from schizonts in our earlier publication detailing the parasite growth dynamic (<https://pubs.acs.org/doi/10.1021/acsinfecdis.1c00262>) and we have included some from these samples in Supplementary Figure 1A. Just for the argument, indeed, *P. malariae* schizonts tend to show a central large pigment with merozoites arranged radially around it however it is not always the case (see https://www.cdc.gov/dpdx/resources/pdf/benchaids/malaria/pmalariae_benchaidv2.pdf).

As for the electronic microscopy images, we compared *P. malariae*‘s ultrastructure to that of published *P. falciparum* TEM and what we called knobs are similar. We agree this could be further be confirmed when a knob molecular marker for *P. malariae* will be available and, as suggested, we called them knobs-like structures. To provide further clarifications, we have added additional TEM images in Supplementary Figure 1B. Accordingly, we have revised the text in manuscript lines 368 and 502.

**Figure 1A: The authors need to provide the parasitemia at time 0 for each sample before showing the parasite staging. A supplementary Figure should show the Giemsa staining of the different samples.**

Thank you for that constructive suggestion. Supplementary Figure 1C has now been included displaying parasitemia at time 0 for each sample and representative pictures of the different parasite stages observed in circulation after Giemsa staining in Supplementary Figure 1A.

**Figure 1D: The TEM images are too small to be informative. The authors must show rings and trophozoites to validate the presence of membrane protrusions at the surface of schizonts. It is premature to call them knobs for *P. malariae*.**

As suggested, we have added ‘knobs-like’ structure in the text. We reckon the TEM pictures displayed in Figure 1D are adequate (size and quality-wise) to prove our point. As the reviewer might probably be aware of, it was described, in the early 80s, that knobs start to appear on the surface of *P. falciparum* infected RBCs at approximately 16 h post-merozoite invasion (Gruenberg J., Allred D.R., Sherman I.W. Scanning electron microscope-analysis of the protrusions (knobs) present on the surface of Plasmodium falciparum-infected erythrocytes. J. Cell Biol. 1983;97:795–802. doi: 10.1083/jcb.97.3.795.), although this timing might vary for other *Plasmodium spp* and remains to be timed. In Supplementary Figure 1B, we have provided other TEM images with trophozoite stages in which knob-like structures are observed.

**Lines 292-301: The interpretation of the evolution of the parasites in the cultures is not necessarily consistent with the observations/data presented. It is highly likely that the authors observed a single invasion event, albeit for only a minor portion of the parasites, but it is likely that a majority of the parasites were not maturing. Again it would be important to show and count the number of mature schizonts with fully formed merozoites.**

We appreciate the comment and we do agree that only a minor portion of parasites progressed *ex vivo* (the % of the different gates are now displayed in Figure 2B) and that usually the case for field isolate parasites.

In fact, we do state in the text (line 303 to 304) ‘*again suggesting completion of one P. malariae asexual stage cycle ex vivo*’. Observing only a single invasion event as the parasites used for this assay were the eluted portion after magnetic sorting (section f in methods). Please also refer to our previous report on the detailed parasite growth dynamic in *ex vivo* culture (https://pubs.acs.org/doi/10.1021/acsinfecdis.1c00262).

All the data used to produce the figures is included in the supporting information excel file. The documents show the number of schizonts and merozoites were counted and, in addition, pictures containing segmented schizonts have been included in Supplementary Figure 1A.

**Figure 2A. The in vitro culture of the negative fraction of *P. malariae* magnetic sorting show a range of trophozoite between 62-82% within 96 hours. Surprisingly the highest level of reinvasion was observed at 48 hours. The authors need to show many examples of the Giemsa-stained parasites at the different time points of the in vitro culture to show the morphology and viability of the parasites.**

Thank you for pointing this out. As recommended, we added pictures of the Giemsa-stained parasites in Supplementary Figure 1A from different time points to show their morphology and viability. As well please refer to the detailed development of *P. malariae* from our previous report (https://pubs.acs.org/doi/10.1021/acsinfecdis.1c00262).

**Figure 2B. The percentages for the events in each gate are missing. The author must show the staging follow-up by flow cytometry with a histogram as presented in Figure 2A for microscopy. Between time 0 and 12 hours the main population of trophozoites disappears meaning that the parasites died because the schizont gate is totally empty.**

Thank you for raising this concern. Please bear in mind these assays were run with the negative fraction of magnetic sorting from 8 *P. malariae* samples of our pool, chosen randomly. As these are field isolates and not culture adapted parasites some of them will certainly not survive from all the experimental procedures we applied. Also we have already published the full development of the parasite that can be seen earlier report (https://pubs.acs.org/doi/10.1021/acsinfecdis.1c00262).

In our study, we aimed to use microscopy, which remains the gold standard technique to assess this parasite, to quantify the different intraerythrocytic parasite stages; while with flow cytometry, we just intended to describe the progression of the parasite but not to quantify the different stages.

**Page 328-345: The very low starting parasitaemias close to the limit of detection by cytometry makes it difficult to interpret the comparative invasion/inhibition assays (Fig3 and Fig4).**

We do agree the task is challenging, however the data are robust and their limitations has been highlighted clearly in the final paragraph of the discussion (lines 453-459).

**Figure 3A. What is the source of** **uninfected red blood cells added for the invasion assay? If they are healthy donors, are they part of the IRB Nº2020-2023/168/CE/FMPOS/FAPH?**

We appreciate your concerns about the ethical compliance of the study. Yes, they are healthy donors and part of the IRB Nº2020-2023/168/CE/FMPOS/FAPH. We have clarified this point by including in the Material and Method section the following text in line 128 to 130 “*The non-infected blood samples came from the Malian Blood Bank and the infected samples were obtained as described below in section b, all complying with the Ethical protocol in place for this study*”.

**Figure 3B. The percentages in the flow cytometry gates must be added.**
These percentages are now added. Please note and see that all the % of new invasions are already shown in Figure 1C where raw data is displayed.

**Figure 3C. What is the limit of detection for infected red blood cells with the flow cytometry assay when the author used uninfected red blood cells as negative control? The parasitemia at 12h are around 0.01% and it is very close to the limit of detection for flow cytometry due to artefact signal associated with reticulocytes, leukocytes or dead parasites with Sybr Green (DNA staining) and a Blue laser for excitation.**

Due to the characteristics and robustness of our assay (described previously in Kariuki SN et al. Red blood cell tension protects against severe malaria in the Dantu blood group. Nature. 2020; or Introini V et al. The erythrocyte membrane properties of beta thalassaemia heterozygotes and their consequences for *Plasmodium falciparum* invasion. Sci Rep. 2022), we can unequivocally determine that those events within the circle are new parasite invasions. Not only because of the Sybr Geen I positive staining but also because they have been able to penetrate a freshly stained RBC (CTFR+) incorporated in the culture.

**Line 347-354: This is the weakest part of the experimental data. The authors cannot conclude with this set of experiments that *P. malariae* can infect reticulocytes, especially as this was only found for a single sample (out of 8). Thus, in the Abstract line 31 in the abstract should be removed, and words “of different maturities” in line 33, and “and that it can invade red blood cells of all maturities” in the Author Summary.**

As requested by three other reviewers that are now satisfied with our clarifications and the results as well the tone used in the text, we defined the selection strategy more strictly (eliminating the most fluorescent CD71 cells as potential false positives, although doing so we may miss some early invasion in very young reticulocytes) and removed background noise from the controls. This is what enabled us to be confident in our findings.

In the abstract we wrote with caution ‘these results suggest’, far from an absolute conclusion, and in the author summary we have modified the text to ‘*including the possibility of invading red blood cells of distinct maturities*’ (lines 44-45).

We believe we have reduced the tone of reticulocyte invasion, and the limitations are clearly, not tacitly, specified in the main text as, for instance, we openly state the limited parasite material (line 453-454) and the low number of samples (line 459).

**Lines 433-452: Given the above comments and those below the claim that this is the first demonstration of reticulocyte invasion by *P. malariae* should be removed, as it is very poorly supported by data that at best hints at this possibility. Chwatt (Ref 48 of the manuscript) had described this as an “exceptional occurrence”. The data presented to support the claim might well result from an artefact of the technique.**

We believe it is harsh to state ‘very poorly supported by data’ when not only us but three other reviewers are convinced by the data provided. We do believe the development of technologies in the last 75 years, as flow cytometry, allows much better resolution than the reference of Chawatt (1948). We have limited the term ‘reticulocytes’ to CD71+ cells, although it has been reported that even some reticulocytes are CD71- (‘Significant Biochemical, Biophysical and Metabolic Diversity in Circulating Human Cord Blood Reticulocytes’ Benoît Malleret, et al. 2013. <https://doi.org/10.1371/journal.pone.0076062>). We are confident we have discarded the possibility of our data being a technical artefact by preparing the appropriate controls, utilizing two different staining approaches and being restrictive in terms of gating. These are the most appropriated dying strategies that could be used in this study given the instruments available and our conclusion is based on outcome from those advanced tools. Despite of that, we have modified the text to ‘*have pointed*’ instead of ‘*have established*’ (line 443) and have added ‘*and encourage further studies in this direction to confirm our findings*’ (line 453).

**Figure 5. To claim any reticulocyte invasion the authors need to add reticulocytes and monitor the expression of CD71 at the surface of the pre-stained reticulocytes before and after invasion. For the only *P. malariae* sample displaying CD71+ Pm-infected RBC, the signal detected with Sybr Green and CD71 PE in the CD71 high population is due to highest autofluorescence of the immature reticulocytes. It is the reason why this percentage is not found with the CD71 APC.**

We have not added purified reticulocytes in blood but measure them directly *ex vivo* in the blood collected which we reason, despite the limitations of losing CD71+ cells as time passes and risking to underestimate the real occurrence of these events, it is a direct way to capture what is happening in nature rather than artificially reproducing a similar, but not equal, context *in vitro*.

**Figure 5C. The author must explain the calculation done to obtain the percentage of infected reticulocytes.**

The % of infected reticulocytes has been obtained directly by calculation of CD71+ cells and either MT+ or SG+ cells removing the background from controls.

--------------------

**Conclusions**

**Reviewer #1: no additional comments**

THANK YOU!

**Reviewer #4:** **The authors provide an interesting set of observations aimed at illuminating some aspects of the biology of the erythrocytic stages of *P. malariae*. This parasite species is little studied and thus, the date is of interest to the community.**

**My main, and major concern, with the twice-revised manuscript are the rather emphatic claims that the authors make (optimized culture conditions, significance of invasion assays, and most glaringly, definitive demonstration of reticulocyte invasion) that are supported by observations on a few samples, furthermore using conditions of parasite development that are far from ideal, and on techniques that are close to their limits of detection, principally because of the low parasitaemias that are inherent to *P. malariae* clinical samples. Moreover, important technical data (such as schemes for calculations) are missing. This is tacitly implied in the text and some of the limitations are explicitly stated in the Discussion.**

Thanks again for your constructive review and comments. Indeed, we are aware the manuscript has been revised twice by three different reviewers, which is a common occurrence, but also the three of them are now satisfied with the quality of our work and the clarifications we have provided. We do not agree with some of the suggestions from reviewer#4 that, even if fair, first, we trust we have acknowledged and addressed, and, second, may undervalue the efforts of this study to further characterize this still largely understudied parasite. Those comments are mainly focused on our claim regarding the invasion of RBCs, which we have now dissected further after our previous publication (<https://pubs.acs.org/doi/10.1021/acsinfecdis.1c00262>), and of RBCs of different maturities for which our results show indications not exempt of limitations. In that sense, we are confident we have taken advantage of a set of advanced tools (i.e. combination of dyes and flow cytometry) compared to microscopy that have provided data-driven claims whose limitations have been emphasized in the manuscript’s discussion section.

--------------------

Editorial and Data Presentation Modifications

**Reviewer #1:**

**- Lines 79, 81 and 96: Remove “that” to make it complete sentence**

The word “that” has been removed from lines 79, 81 and 95.

**- Line 164: “every” not “each”**

Edited in line 167.

**- Line 326: “Evaluate”, not “evaluating”**

Corrected in line 329.

**- Line 341: remove “allowed us to quantify all three enzymes”**
Removed in line 344.

**- Line 353: replace “was confirmed with the two approaches used” with something more specific, like “showed invasion of reticulocytes”**

Amended in line 355 as suggested.

**- Line 395-396: This sentence feels very long. I would edit to “…it is likely to employ alternative mechanisms to gain entry into RBCs, as well. A model showing potential invasion pathways is shown in Figure 6.”**

Thanks for the suggestion. This sentence has been edited (lines 397-398).

**- Line 396: I would modify this sentence to “In alignment with this hypothesis, a report from the last decade described an unconventional invasion pathway utilized by Colombian and Peruvian field isolates that was resistant to simultaneous pre-treatment of RBCs with these enzymes.”**

Thanks for the suggestion. This sentence has been modified (lines 398-401).

**- Line 403: change “presence” to “identity”.**

Edited in line 404.

**- Line 424-425: remove “displaying also the effects of potential blocking agents”.**
Removed in line 426.

**- Line 441: remove “Contrary to existing knowledge,”**

Removed in line 442.

**- Line 449: “parasite” not “parasite’s”.**

Corrected in line 449.

**- Line 449: remove “Despite our findings” and break up sentence into 2: “Capturing these events is difficult due to a combination of factors, such as low parasitemias with reduced number of reticulocytes in the samples and a narrow detection window. These challenges have limited our ability to quantify precisely the frequency of this phenomena in *P. malariae* wild parasites.”**

Thank you for rewriting that sentence. Words are removed and sentence edited in lines 449 and 452.

**Reviewer #4: (No Response)**

--------------------

**Summary and General Comments**

**Reviewer #1: Manuscript is significantly improved from the previous versions!**

THANK YOU!

**Reviewer #4: The data presented in this manuscript are of interest, but these are insufficient to support some of the conclusions and claims presented (see above).**

## We have made our best attempt to address your comments and discussed the best of our knowledge, finding obtained from the results. We believe it is a significant progress within the field due to the inherent and challenging characteristics of *P. malariae* (i.e. seasonal appearance, low parasitemias, common co-infections with other *Plasmodium spp* and the absence of continuous *in vitro* culture). We have carefully drawn our conclusions and have been cautious all level by highlighting the current limitations of our study to encourage further investigation.

--------------------

Reviewer #1: Yes: Kathleen Dantzler Press

Reviewer #4: No
