# Supplementary material for: Malian field isolates provide insight into Plasmodium malariae intra-erythrocytic development and invasion
Source: PLoS Negl Trop Dis. 2025 Jan 6;19(1):e0012790. doi: 10.1371/journal.pntd.0012790 (PMC11735006; doi:10.1371/journal.pntd.0012790)
Supplement: Attachment — Submitted filename: 240524_Dao et al PLoSNTD_Responses to reviewers.docx [file pntd.0012790.s003.docx]

When you are ready to resubmit, please upload the following:

[1] A letter containing a detailed list of your responses to the review comments and description of the changes you have made in the manuscript.

[2] Two versions of the revised manuscript: one with either highlights or tracked changes denoting where the text has been changed; the other a clean version (uploaded as the manuscript file).

Important additional instructions are given below your reviewer comments.

Please prepare and submit your revised manuscript within 60 days.

***********************

Reviewer's Responses to Questions

**Methods**

**Reviewer #1: Objectives are clear. The study is presented as an open-ended characterization with no clear testable hypothesis. To improve this, the authors could add their hypotheses around what they expected to be similar vs. different from P. falciparum/vivax.**

This suggestion has been addressed in the last section of the results and displayed in Figure 6 (lines 352-361). Please, see comments from Reviewer 1 in Conclusion section for more details.

**Sample sizes are small, but seem reasonable given the challenges of acquiring P. malariae isolates in the field.**

**Statistical analyses seem appropriate.**

**No concerns.**

**Reviewer #2: In this work, clinical samples were obtained from de-identified donors who had tested positive for P. malariae in a screening study. PCR was used to identify samples with mono infections with P. malariae, which were then investigated for clinical characteristics, parasite phenotypes, and assessment of potential invasion assays. The invasion assays required adoption of a flow cytometry-based method to measure low parasitemia, which has been used in falciparum and vivax studies. The sample sizes were sufficient overall, but in some figures the exact n that was used was not clear, and did not seem to match the number of data points visualized in the figures. Additionally, in several bar graphs it is not clear what is being plotted.**

Thanks for the comment. We have incorporated clear n values in all the legends of the figures and explicitly stated what they refer to.

As for the bar graphs, Figure 1A shows the % of parasite stages in the smears read at withdrawal (error bars show SD); while Figure 2A shows parasite progress *in vitro* after removal of MACS purified schizonts that were used for invasion assays.

**Reviewer #3: The methods are appropriate for this paper and the authors address the limitations in the Discussion due to the use of patient samples.**

--------------------

**Results**

**Reviewer #1: Results are clearly presented. I would suggest making figure titles more specific, indicating a clear conclusion rather than general characterization. Some specific suggestions for figures, particularly Figure 4, are below. I would suggest including exact p-values, not just asterisks.**

Thanks for the suggestions, titles of figures 3 (line 513), 4 (line 522) and 6 (line 538) have been modified accordingly. p-values have been incorporated in Figures 3 and 4.

**I would suggest substantial revisions to Figure 6. The current figure seems more appropriate to a review article.  Rather than summarizing which ligands are susceptible to the different enzymes according to the literature, I think it would be more useful to make a model with proposed invasion pathways for P malariae based on the results in this paper.**

Thanks very much as this suggestion has made us realize that we could have done better here and dig deeper in our own findings, come up with an hypothesis even if it is not completely conclusive due to the limitations of the study. Please see full description of our hypothesis at the end of the result section (lines 352-361).

**Reviewer #2: (No Response)**

**Reviewer #3: Yes, the results are presented clearly and the figures are of sufficient quality for clarity.**

--------------------

**Conclusions**

**Reviewer #1: Conclusions are more negative results than positive, but are important contributions to the field given the large gap in knowledge in this area.**

**The discussion seems to imply a bigger importance for reticulocytes than the results suggest- I would de-emphasize this a bit and discuss more the implications/hypotheses underlying why reticulocyte invasion is observed in a small number of individuals but not majority.**

Although we find this result relevant and interesting we have limited its impact in the manuscript (see lines 438-446) and commented on the reasons why we only observe them in a small number of individuals (see lines 446-449).

**Why is there no impact of CD35, CD147, CD235a antibodies if these are sensitive to 1 or more of the enzymes and there were significant differences with the enzymes? This point, and more generally the integration of the antibody and enzyme data, should be discussed in Discussion.**

The absence of impact of specific antibodies against these three receptors could be explained with several hypothesis. First, there are no annotated orthologues neither for PfRH4 (CD35 ligand) nor for PfRH5 (CD147 ligand) nor for EBA175 (CD235a) in *P. malariae*. Thus, there was no surprise to confirm no specific effect in our study, although the justification to use them was that, if any blocking effect would have been observed, they could have pointed out the existence of novel *P. malariae* ligands benefitting from these receptors. Second, the utilisation of monoclonal antibodies, as highlighted in the text, might not target/block the specific ligand-receptor molecular interactions. Third, the apparent incongruencies with the enzyme data could be explained, as shown in the updated version of Figure 6 and detailed further in the discussion section (see lines 400-405), with the availability of other receptors, with different enzymatic profile, in each stage of the process. That could make the parasites counterbalance the effect of individual enzymes and penetrate the RBC using alternative ways. Due to the limited number of assays that could be run with each sample, we did not test combinations of different blocking agents that could have addressed this question better.

**A major limitation, which the authors address, is the low percentage of new invasions and therefore difficulty in assessing significant changes. The decision to count a higher number of events as a result seems reasonable.**

Thanks for the comment. We appreciate the reviewer acknowledges the challenges of our study.

**The public health relevance could be highlighted more. For example, the impact of anaemia on morbidity could be mentioned, and the incidence of P malariae in Mali could be discussed.**

As requested, the impact of anaemia due to malaria infections has been commented in line 61 and the incidence observed in our study is in agreement with previous observations as mentioned in line 367.

**Reviewer #2: (No Response)**

**Reviewer #3: Yes the conclusions are supported by the data presented. There are some caveats to using monoclonal antibodies against red blood receptors to examine *P. malariae* invasion and I have highlighted this to be addressed in the discussion.**

--------------------

**Editorial and Data Presentation Modifications**

**Reviewer #1: The title seems a little awkward and not as compelling as it could be. I would suggest something like “Malian field isolates provide insight into Plasmodium malariae intra-erythrocytic development and invasion”**

Thanks for the comment. We have entitled the manuscript as suggested as it indeed captures better the impact of the study.

**Line 61 – I would replace “although the underlying mechanism are not yet known” with “leading to substantial impacts of morbidity” or something that highlights the clinical impact of glomerulonephritis/anemia**

This suggestion has been incorporated in line 61

**Clarify in methods that 45 individuals recruited were positive for P. malariae (not just Plasmodium-infected as it says in line 138). Clarify inclusion criteria – is diagnosis of P. malariae by blood smear? Or qPCR?**

A clarification has been provided in line 143 in methods, and line 274 in results. 45 individuals were recruited as P*. malariae* positive by microscopy. 30 of those resulted in qPCR *P. malariae* monoinfections and only this subset of samples was analysed further in subsequent assays.

**Clarify complete media components. Are culture conditions similar to Pf? Are these conditions well-established for P. malariae?**

Thanks for pointing this out. Media components for *P. malariae* have been established, by co-authors of this study, for *ex vivo* cultures (see doi: [10.1021/acsinfecdis.1c00262](https://doi.org/10.1021/acsinfecdis.1c00262)), but not for continuous *in vitro* culture, and differ from medium used in *P. falciparum* culture only in the % of glucose content. The reference has been explicitly added in line 164.

**Add antibody dilutions used**

For this study, given the limited number of conditions that could be tested with a given sample due to low parasitaemias, we used a unique reference concentration for all the antibodies tested of 50µg/mL.

**Line 286 – what does the existence of knobs suggest about possible surface ligands?**

We have referred to this point in the discussion (lines 377-381). There has been no KAHRP (Knob-associated histidine rich protein) nor *var* gene orthologues annotated in *P. malariae*. However, we have identified several orthologues of proteins that are involved in protein trafficking to the RBC membrane as described elsewhere (<https://doi.org/10.3390/tropicalmed8070353>) that might play a relevant role.

**Figure titles – change to make more specific and state the conclusion of figure, e.g. for figure 2, “P. malariae replicates and matures in vitro” instead of “Characteristics of P. malariae in vitro”**

Thanks again for this comment as it highlights better the results displayed. This has been changed in Figure 2 (line 502), 3 (line 515), 4 (line 524) and 5 (line 533), and Table 2 (line 501).

**Line 319 – enter not entry**

Edited in line 321

**Line 327 – latter not later**

Edited in line 329

**Line 330- I would use phrase other than a “battery”**

Edited in line 332

**Figure 2 - does n=178 refer to individual cells? how many patients is this from?**

This has been clarified in the main text in line 290 (data from 8 patients) and in the legend of Figure 2 (line 503).

**Figure 3 - are cells gated at all prior to SG/CTFR gate shown? It would be worth having a singlet gate to confirm you are not just measuring autofluorescence.**

Yes, cells are gated prior to SG/CTFR indeed. We preferred to display the full gating strategy in Figure 5 as the results could raise more concerns. As shown there (line 532), two prior gating steps were performed in all samples collected by flow cytometry to ensure appropriate data analysis. First on the FSC-A vs SSC-A channels gating the general population, and then in the FSC-A vs FSC-H channels to define the singlets.

**Figure 3C- include N.**

Thanks for pointing this out. The figure includes now the N (line 528)

**Figure 4 – It doesn’t seem necessary to me to include individual graphs and to show the Control at 100% so many times. I would recommend including all antibodies on the same graph, removing the control dots, and indicating on the axis that the values are normalized.**

This has been modified in Figure 4 as suggested, displaying all the antibodies in just one graph (line 524).

**Figure 4C- what are the gray dots in the background? can these be removed?**

There is no figure 4C and no gray dots are shown in the background of any figure so, apologies, we are not able to identify what should be modified.

**Figure 5B – why is it needed for quadrant gates to not be straight? Is the compensation definitely correct?**

Indeed, this was a point largely discussed among the team and with the head of the flow cytometry facility at MRTC. Straight lines in the plots would have incorporated a larger number of false positives and further compensation would have missed the true positives. Due to the assay limitations, stated openly in lines 448-451, we decided this to be the most accurate manner to display and analyse our results.

**Figure 5C – the green dot doesn’t look that different from 0 – is this confirmed reinvasion? It looks to me like only the red dot is clearly invading reticulocytes.**

Our point here was to show that there was a sample clearly showing reticulocyte invasion (in red) regardless of the approach, and, as this is the first time reticulocyte invasion has been shown for *P. malariae*, another one that could be a positive (in green) but for which a very low number of events had been captured. This has already been corrected in Figure 5C and colouring has been harmonised having left only the clear sample in red (line 532).

**Discussion – wording and sentence structure could be improved generally.**

We have attempted to the best of our knowledge to correct this across the manuscript.

**Figure 6 – cite at end of Results section.**

As suggested, Figure 6 has been edited and incorporated at the end of the results section (line 355).

**Line 402 – expand more on fam-l and fam-m genes. Also clarify why you expect a homolog of Rh5 to be more likely to be important rather than a new unidentified receptor. Including experiments that target these proteins would definitely make the paper significantly stronger, but I realize that creating new antibodies may be prohibitive.**

As requested, we have expanded on fam-l and fam-2 genes in lines 425-427.

As for Rh5, that is a very fair point. As an open-ended study with no prior hypothesis, we focused on the first characterization of *P. malariae* invasion at molecular level based on existing knowledge in other *Plasmodium spp*. Identifying new ligands from the parasite side and/or novel receptors in the RBC is within our future lines of research but beyond the scope of this study.

We absolutely agree with the latest point from the reviewer, producing specific antibodies against potential targets would significantly enhance the impact of our study, however that would involve a significantly larger investment both time and budget-wise that unluckily, this time, we could not afford.

**Line 433 – Rephrase – for example “furthermore, the study was limited to a small number of samples, which necessitates additional studies…”**

This has been rephrased as suggested in line 458.

**Line 436 – “antimalarial drugs” instead of “antimalarials”**

Edited in line 461.

**Reviewer #2: 1. The information on the parasite morphologies and life cycle stages identified in the samples adds to our knowledge. However, it would also be helpful to have some information about the parasitemia in these samples. This should ideally be added as a panel to Figure 1.**

Thanks for the valuable comment; parasitemias of all samples are available in Supplementary file 1.

**2. Given the low parasitemias observed in Figure 3C, for Figure 4 the raw parasitemia data should be made available in a supplemental file to assist with understanding the biological significance.**

As for point 1, parasitemias of all samples have been made available in Supplementary File 1.

**3. For the invasion assays, what was the initial starting parasitemia that was added?**

The initial starting parasitemia for invasion assays was not estimated. The practical reason for that is that after concentrating *P. malariae* late stages with MACS columns from 5mL of blood withdrawn from patients, the pellets obtained were of 1µL or less. To maximise the usage of that amount was challenging so no smears were taken nor flow cytometry used to estimate the initial parasitemia.

**Reviewer #3: Required changes:**

**1. In Table 2 and Figure 6, it is stated that the enzyme treatment requirement for PvRBP2b is not known. This is incorrect as in Fig. S1 of Gruszczyk et al Science 2018, it was shown that PvRBP2b binding to reticulocytes was sensitive to trypsin and chymotrypsin but resistant to neuraminidase. This information should be updated in Figure 6, Table 2 and all the corresponding text within the manuscript.**

Apologies for having provided incorrect information. This has been updated as suggested in Figure 6 and Table 2 (see lines 540 and 513, respectively).

**2. In Table 2, CD71 is referred to as Transferrin. This is incorrect and CD71 should be referred to as Transferrin Receptor 1.**

Thanks for pointing this out, this has been corrected in Table 2 (line 513).

**3. In Table 2, for PfRh4, please also add reference Tham et al, Infection and Immunity, 2009 PMID: 19307208 as this was the first paper to describe the enzyme profile for the binding.**

As for comment 2, this has been corrected in Table 2 (line 513)

**4. Line 95 to 96 in Introduction states “Secondly, the P. vivax RBP2b that interacts with one of the key reticulocyte markers,the transferrin receptor (CD71)(32)(33)(34)(35).” Most of the references for this statement are incorrect and should be changed to (Gruszczyk et al Science 2018 PMID: 29302006, Gruszczyk and Huang et al Nature 2018 PMID: 29950717, Chan et al, Nature Communications 2021PMID: 33750786, Chan et al, Cell Microbiology 2020, PMID: 31469946)**

Many thanks for this comment as it made us realise that we had a reference numbering issue. It has been corrected as it appears in line 96.

**5. In Introduction, line 103 to 105, “One of those, the RH5 kite-shaped fold, is known to be also present in RBP2a in P. vivax and may be a key conserved structure for the binding capabilities of all Rh and RBP genes (41). Please also add Gruszczyk et al Science 2018 and Gruzczyk and Huang et al, Nature 2018 as references as PvRBP2b also shows the same structural fold as PfRh5.**

Thanks for the suggestion, as those were ground-breaking papers in research on how *P. vivax* invades RBCs and were not clearly highlighted. The two references have been incorporated in line 105.

Minor consideration:

**1. Under Molecular Players in P. malariae RBC invasion: The authors provide a summary of potential orthologues based on existing genome annotation of Plasmodium genomes. It would be interesting to run an Alphafold/Foldseek comparisons of the standard structural scaffolds of PvRBP/PfRh family and PfEBA family of proteins to observe what is present structurally in the P. malariae genome. This is only a suggestion and if not done, it does not impact the novelty of this paper.**

We appreciate this suggestion. In fact, the utilisation of Alphafold/Foldseek is in alignment with our future efforts to identify novel *P. malariae* potential ligands.

**2. More recently, CD98 was observed to be a receptor used by P. vivax potentially through binding of PvRBP2a. Can this be updated in the relevant tables and figures? No required additional experiments.**

Sure, we have incorporated that in Table 2 and Figure 6 as suggested (see lines 540 and 513, respectively).

**3. Add an additional reference to “Second, the reticulocyte binding protein (RBP) family that includes the P. yoelii 235-kDa rhoptry proteins, the P. vivax reticulocyte binding proteins (PvRBP-1 and -2), and the P. falciparum reticulocyte homology proteins (PfRH1, PfRH2a, PfRH2b, PfRH4, and PfRH5) (25)(24)”. Please add Chan et al, 2020 Cell Microbiology PMID: 31469946 as it also provides a more up to date review of the PvRBP family of proteins.**

We have added the reference as suggested (line 83).

**4. For Figure 2, if already available, would it be possible to also have figure panels showing the presence of rings at 96 hr to show that there has been a reinvasion process to go together with the flow cytometry plots?**

Thanks very much for the suggestion, although, unfortunately those pictures are not currently available.

**To be addressed in Discussion:**

**The authors use a panel of antibodies against known receptors and show that the addition of those antibodies does not block P. malariae invasion. From the panel chosen, many of the antibodies are monoclonal antibodies that have not been shown to disrupt the known invasion ligand-receptor interaction or parasite invasion. For example, anti-CD35 (BioLegend, 332402) and anti-CD71 (Miltenyi Biotec, 130-124-327 (Clone REA902) have never been shown to block P. falciparum or P. vivax invasion. In particular, there is no anti-CR1 mAb known to block invasion and also only 5 out of 9 anti-CD71 mAbs tested in Gruszczyk et al Science 2018 were shown to block reticulocyte binding. Therefore, it would good to address these caveats in the Discussion and propose some ways forward for future studies that do not include untested monoclonal antibodies:**

**a.    	Most monoclonal antibodies chosen in this paper have not been shown to block P. falciparum and P. vivax invasion.**

**b.        May be important to initially use polyclonal antibody against the receptors if the binding sites for P. malariae is not known.**

**c.        If available, the use of recombinant soluble receptors such as soluble CR1 (Tham et al, PNAS 2010) to understand the effect of inhibitor.**

We appreciate this very fair point as we also realize that it is one of the limitations of our approach. Together with the fact of only being able to use a single concentration of the antibodies in a given sample due to low parasitemias. We have addressed these limitations in the discussion. We have also included the suggestions provided for future efforts and the reference for soluble CR1 (line 421).

--------------------

Summary and General Comments

**Reviewer #1: This study uses Plasmodium malariae field isolates to characterize the invasion and development of this parasite. The authors effectively put their work into the context of what is known about intraerythrocytic development of P falciparum and P vivax. They develop an assay to assess in vitro invasion and development by the parasite, and to probe the role of different receptors of interest. Overall, given that P malariae is an understudied parasite with very little known about its biology, the contributions by Dao et al. are novel and valuable to the field. The manuscript could be strengthened with some edits to Figures 4 and 6 and editing the language to be clearer.**

As suggested, Figures 4 and 6 have been modified (lines 523 and 539, respectively) and language has been reviewed by two native speakers.

**Reviewer #2: In their study, Dao et al investigate clinical samples of Plasmodium malariae, an under-studied species of Plasmodium parasites that can cause malaria in humans. The lack of an in vitro culture system for P. malariae and its tendency to occur as a co-infection have limited its use in experimental studies. The demonstration of an assay to study invasion of P. malariae from clinical isolates in vitro is an important advance for our understanding of this organism.**

**1. A primary finding from the analysis of the clinical characteristics of the samples was that P. malariae is associated with anemia.  However, the data in Table 1 do not provide any indication of the level of anemia in the general population (not infected with P. malariae). Given that anemia is often multi-factorial, the prevalence of anemia in the villages/age groups from which the samples were drawn should be reported.**

Thanks for raising this point. However, the prevalence of anemia in the local population is not regularly assessed. Thus, here we focus on the raw observations without concluding a relationship between *P. malariae* infections and anemia detection in the patients participating in the study.

**2. For several figures, there is not a clear indication of the sample number used and how it relates to the presented data. For Figure 1A, what is the sample number and what do the error bars represent?**

Apologies if the sample number was not clear in several figures. This has been clarified in the legends of all figures.

**For Figure 2A, what is meant by n=178?  The study reportedly only used 8 samples for in vitro culture – please explain n=178.   Similarly, why is n=65 for Figure 2C? Is this data from 65 schizonts from a single donor? Please clarify.**

Again, thanks for the question. We have modified this n to 121 that is the number of slides read, including all time points for 23 samples (line 503). We had included originally 178 as we had taken extra time points in some of the samples that have not finally included in the figure.

In Figure 2C, we have corrected the numbers as they were in fact 55 schizonts, in which the number of merozoites could be observed, from 11 different samples (line 508).

**For the invasion assays presented in Figure 3C, please include the sample number (n). The text implies that parasitemias were quantified in duplicate, but what is plotted on the graph in 3C?**

This has been clarified in the legend of the figure (lines 520-521).

**In 4A, legend indicates n=3, but what is this number referring to?  Most of the graphs seem to show 4-5 data points.**

This has been clarified in the legend of the figure (lines 527-528).

**In Line 339, percent reduction in invasion is listed (referring to Figure 4B).  Please clarify what these numbers refer to: is it the mean reduction for the 6 samples?**

Yes, indeed this is the mean reduction for the 6 samples.

**3. For figure 1A, what is the explanation for the low percentage of rings reported for the samples, when Figure 2A demonstrates a high percentage of rings at 0 hours?**

Thanks for the question. Numbers for figure 1A were obtained from thin smears observations taken from samples upon reception. While numbers for Figure 2A were acquired after having magnet-purified the samples. Therefore, the most likely explanation for the differences observed would be that having removed mostly, but not all, schizonts and potentially some trophozoites, the percentage of rings would automatically be augmented.

**4. In Figure 4, 8 different antibodies are tested for their ability to block *P. malariae* invasion.  Are these the same clones/concentrations that have been shown to impact Pf/Pv invasion in the literature? This would be important information to support the conclusion that P malariae entry may not rely on any of these receptors.**

We agree that this is a limitation of our study. As a blind study and due to the variability of certain antibodies against known receptors (as referee 3 also pointed out, doi: 10.1126/science.aan1078), we aimed to use antibodies which were well known to bind RBC receptors. We have stated clearly those limitations in lines 417-421.

**5. The conclusion that P. malariae is capable of invading reticulocytes would be important, but the evidence supporting this conclusion is not sufficient because Figure 5 is difficult to interpret. At a minimum, the flow cytometry plots from the two samples reported to demonstrate invasion in reticulocytes should be shown. However the conclusion would be better supported by using FACS to sort SG+/CD71+ cells to show that these are indeed infected by P. malariae.**

To facilitate interpretation, we have just highlighted the one sample (EEF482) that has been positive with both approaches, which is displayed in Figure 5. We agree with the reviewer that the number of positive samples is limited to one, however,  given the difficulties of capturing this event (i.e. *P. malariae* monoinfections, that are usually confirmed by qPCR 24 hours after withdrawal, with low parasitemias and reduced % of reticulocytes), and being this one the first time that has been observed, we do believe it is worth showing the data and opening the door to further studies in this direction.

We have slightly de-emphasised the importance of this finding in the discussion section (lines 448-451).

Sorting DNA+/CD71+ cells and checking whether they are certainly *P. malariae* RBCs is an excellent idea. However, it would imply having a sorter in the facilities at MRTC, which was not available at the time of our study.

**Reviewer #3: The manuscript by Dao et al describes characteristics of P. malariae infection/invasion from patient samples and a one cycle culture assay which they have established. P. malariae is one of the six malaria parasites that infect humans and is very understudied. As global efforts move towards malaria elimination, it is paramount that the basic biology of how P. malariae infects human red blood cells is understood. As there is no existing culture system for P. malariae, the authors are using patient samples which provide an important snapshot of what is occurring in human infections. In addition, to the best of my knowledge, the authors also provide evidence for an in vitro short-term culture that shows at least one cycle of reinvasion which is an important advance in the field. They proceed to determine if the receptors, surface expression properties (via enzyme treatment of RBCs) and types of RBCs are similar between what is known for P. vivax and P. falciparum.**

**This is an impressive line of questioning in light that it has been done with patient samples and therefore this paper provides some important insights into P. malariae invasion.**

--------------------

Reviewer #1: Yes: Kathleen Dantzler Press

Reviewer #2: No

Reviewer #3: Yes: Wai-Hong Tham

Figure Files:

While revising your submission, please upload your figure files to the Preflight Analysis and Conversion Engine (PACE) digital diagnostic tool, https://pacev2.apexcovantage.com. PACE helps ensure that figures meet PLOS requirements. To use PACE, you must first register as a user. Then, login and navigate to the UPLOAD tab, where you will find detailed instructions on how to use the tool. If you encounter any issues or have any questions when using PACE, please email us at figures@plos.org.

Data Requirements:

Please note that, as a condition of publication, PLOS' data policy requires that you make available all data used to draw the conclusions outlined in your manuscript. Data must be deposited in an appropriate repository, included within the body of the manuscript, or uploaded as supporting information. This includes all numerical values that were used to generate graphs, histograms etc.. For an example see here: http://www.plosbiology.org/article/info%3Adoi%2F10.1371%2Fjournal.pbio.1001908#s5.

Reproducibility:

To enhance the reproducibility of your results, we recommend that you deposit your laboratory protocols in protocols.io, where a protocol can be assigned its own identifier (DOI) such that it can be cited independently in the future. Additionally, PLOS ONE offers an option to publish peer-reviewed clinical study protocols. Read more information on sharing protocols at https://plos.org/protocols?utm_medium=editorial-email&utm_source=authorletters&utm_campaign=protocols
