# Supplementary material for: Malian field isolates provide insight into Plasmodium malariae intra-erythrocytic development and invasion
Source: PLoS Negl Trop Dis. 2025 Jan 6;19(1):e0012790. doi: 10.1371/journal.pntd.0012790 (PMC11735006; doi:10.1371/journal.pntd.0012790)
Supplement: Attachment — Submitted filename: 240903_ManuscriptReview_Respond to reviewers.docx [file pntd.0012790.s004.docx]

**Rebuttal letter**

**Dear Editor**

**Please find the revised version of our manuscript submission (PNTD-D-24-00247R1) - [EMID:095586fd8131d1af] entitled “*Malian field isolates provide insight into Plasmodium malariae intra-erythrocytic invasion and development*”.**

We would like to thank the reviewers for their constructive comments. We have thoroughly examined the manuscript based on the instructions given and their suggestions/concerns below. We have made our best to address them all and our point-by-point responses have been included with this letter. Please note that all line numbers are referred to the ‘clean’ version of the article, with all changes accepted.

**Reviewer's Responses to Questions**

**Methods**

**Reviewer #1:**

**No major comments. I did wonder about the single concentration chosen for the antibodies being tested. Why was this concentration chosen and is it possible you would have seen different results if a different concentration was used? If this concentration has been used by other studies, those should be cited in the methods.**

Thank you for requesting clarification on this point. We think there are three main factors that justify the usage of a single antibody concentration, all based on maximizing the usage of precious *P. malariae* monoinfections collected in a narrow seasonal window while keeping consistent our approach across the entire duration of the study:

i) the limited number of assays that could be run with each sample due to low parasitaemias;
ii) the concentrations at which all the purified antibodies used in this study were purchased at (either 1mg/mL or 0.5mg/mL);
iii) the low final culture volume in each well (50µL).

With no possibility to dialyze or increase the concentration of the antibodies, and given the fact all the antibodies were resuspended in saline solution, we decided to use the highest concentration possible for all antibodies, without going any higher than 10% of the final culture volume. A negative control without antibody but containing the same amount of saline solution was prepared for each experiment.

To our knowledge, there have been several reports showing effective growth inhibition using antibodies with lower concentrations than 50µg/mL (see Tran et al. 2014 ([J Infect Dis.](https://www.ncbi.nlm.nih.gov/pmc/articles/PMC3923542/) 2014 Mar 1; 209(5): 789–798), Douglas et al. 2014 ([J Immunol. 2014 Jan 1; 192(1): 10.4049/jimmunol.1302045](https://www.ncbi.nlm.nih.gov/entrez/eutils/elink.fcgi?dbfrom=pubmed&retmode=ref&cmd=prlinks&id=24293631)), Tham et al. 2010 (Proc Natl Acad Sci U S A. 2010 Oct 5;107(40):17327-32), Iqbal et al. 1997 (Parasitology. 1997 Apr:114 ( Pt 4):317-24)). All these reports made us confident on the utilization of this concentration, although, it is likely that higher concentrations might have provided a different result we state in the text ‘at the single concentration tested’ (lines 337 and 409) so we do not over interpret our results.

**Reviewer #2:**

**The objectives of the study are clearly articulated, and the study design using patient samples is appropriate to investigate the biology of *P. malariae* since this organism cannot be readily cultured.  Sample sizes seem appropriate, though there are inconsistencies in the sample size reporting, which should be corrected.**

Thanks for your remarks and thorough reading of the manuscript. We have addressed all the inconsistencies identified and apologize for the confusion. Depending on their availability, we used a different number of samples for each experiment, as explicitly mentioned in the corresponding section, that are now clearly numbered:

- The total number of individuals determined by microscopy and enrolled in the study was 45 infected with *Plasmodium malariae* and who met the inclusion criteria (lines 139 and 267).
- For clinical evaluation, we used 45 *P. malariae* infected individuals, determined by microscopy. For 28 of those, the haemoglobin levels were also measured (Table 1, line 492).
- The number of *P. malariae* samples confirmed to be monoinfections by qPCR, as described in methods, was 30 and only subsets of these were used for subsequent assays (lines 276 to 279).
- *P. malariae* invasion and development dynamics were monitored by light microscopy taking smears from 23 different *ex vivo* cultures across the time. The number of smears read for each time point is explicitly shown in the legend in Figure 2A (lines 508-509). Eight cultures were also evaluated by flow cytometry (lines 168, 295 and 511).
- Nine samples were used to optimise the assay to quantify *P. malariae* RBC invasion (lines 331 and 528).
- At least four samples were utilised to evaluate the blocking effect of each of the antibodies tested (lines 336 and 533 to 536); while six samples were used with the different enzymes (lines 343 and 537).

**---------------------------------------------------------------------------------------------------------------------------**

**Results**

**Reviewer #1:**

**The figures and discussion of results are much improved. The figure/section headings and inclusion of p-values and sample sizes are also improvements. My one remaining concern is Figure 6. The accompanying paragraph describing possible hypotheses for invasion is an interesting discussion of the implications and knowledge gaps associated with this work. However, the figure does still imply that a lot more is known or backed up with evidence than the results from this paper indicate. The explanation of possible redundancy across pathways makes sense but that still doesn’t provide any clear support for those proteins being definitively involved. Could you adjust the figure so that it 1) does not include proteins where blocking did not impact invasion in this paper (unless you have strong reason to believe this was because of redundant pathways) and 2) highlights proteins that have known sensitivity to the enzymes but were not tested in this paper with question marks? Alternatively, since this is more speculative than backed up with data, it might also be worth considering removing the figure and moving the hypothesis paragraph to the discussion.**

Thanks for pointing this concern out. As recommended, we have moved the hypothesis paragraph to the discussion (lines 425 to 432). However, we have decided to leave the figure in the discussion to show a model of hypothetical *P. malariae* pathways but not in the results section. We agree not all the proteins included in the figure have been tested in our study and the blocking effect of certain antibodies might be at a higher concentration than the one we tested; although we are confident the figure provides valuable information from our data and facilitates de visualization of the potential *P. malariae*-RBC molecular interplays. We have adjusted the figure highlighting the proteins for which no antibody was tested against, but still displaying other potential actors in this complex interplay to suggest potential avenues for future research.

**Reviewer #2:**

**The figures and analysis presented match the analysis plan of the study. However, there are several aspects of figures that are unclear, and sometimes there is inconsistency between the text, legends, and/or methods.**

**1. For Figure 1B, the “n” should be 23, not 11, because data from all 23 samples are plotted.**

Thanks for the comment. This has been corrected in the text (line 500).

**2. In Figure 2, the number of samples used is still not clear.  The results section indicates that 8 patient samples were used, but the legend says n=23.**

Apologies for the confusion. The legend has been corrected and the number of samples clearly detailed (lines 507 to 515).

**3. In Figure 2A, the authors show the changes in parasite stages over time in vitro culture, but there is no information about whether the percentages presented are additive for all samples, average, or something else. This does not allow for any information about the variability between samples. A more informative visualization of these data would be to plot the data for each sample, with points for each stage (represented by different shapes) and each sample. Thus, at each time point a total of 23 x 3= 69 points should be plotted (or 8x3= 24 points if indeed the sample number was 8 and not 23).  Alternatively, the data could be plotted as a bar graph as in current version, but the data included data should be from each sample or average for all samples (with standard number of cells counted per sample indicated), and error bars/ standard deviation should be provided.**

Thanks for the suggestions. A new graph of figure 2A has been drawn up. As suggested, a bar graph with the average for all samples with error bars showing standard deviation has been plotted.

**4. In response to a concern about the discrepancy between the ring stage parasitaemias shown in Figure 1A and Figure 2A, the authors suggest in their response that the samples in Figure 2A were magnet purified, which might enrich for rings. However, there is no indication of magnet purification having been performed for the data presented in Figure 2 in the methods, legend, or results sections. Indeed, the experimental method implies that samples went straight from isolation to culture without any manipulation beyond washing. This needs to be reviewed and clarified**.

We apologies if this had not been stated clearly in the text. Having included in the methods section ‘upon reception of positive samples detected by microscopy, in all samples, *P. malariae* late stages were enriched using LS magnetic columns’ (line 186), we thought to have addressed this comment. However, as it has not been the case, we have also added in the results section where Figure 2A is mentioned, ‘we used the eluted portion after magnet purification of twenty three patient samples, aiming to enrich early stages’ in the text (lines 292-293) to clarify it further.

**5. In Figure 2B, the legend indicates NV, R, T, and S, but these notations are not found in the revised figure.**

Thanks for the comment. The figure now incorporates the gate names matching the legend (line 505).

**6. In Figure 3C, the legend indicates n=18, but the text in the results section indicates that the sample number was 9 and that each sample was quantified in duplicate with the average parasitemia plotted.  The graph itself in figure 3C appears to show somewhere between 10-18 data points (~16 lines connecting the two time points are seen).   Please correct these inconsistencies in the data, text, and legend.**

Thanks for pointing this out. It has been corrected in the legend (line 528) and on the figure. Nine samples in duplicate as written in the text (line 331) is correct with the average plotted in the figure.

**Reviewer #3**
**1. Figure 1 B) the original figure listed n=28. The figure legend now states n=11 but there are clearly more data points in figure B.  It appears that the 12 specimens had no schizonts.  To be consistent the n should be listed as 23 to include the samples that had no schizonts as is the case in panel C where the samples with no gametocytes are included in the “n” number.**

Thank you very much for the comment. This correction has been incorporated in the legend of the figure (line 500).

**2. Figure 2A) the figure lists 23 samples and n=121 for smears.  If a smear was done for each sample at all 7 time points the number of smears should be 161.  Further confusion is caused by the text which states that “To investigate the dynamics of parasite development, we used eight patient samples, incubated them under standard culture conditions and monitored them every 12h using light microscopy.  This must be clarified.**

Apologies for the confusion. The calculations are correct to end up reading 161 smears, however, not all the smears were taken and not all the smears were of sufficient quality and, therefore, exploitable. We ended up counting the different stages in 121 smears from 23 samples at the different time points (see legend Figure 2, lines 508-509).

**3. In Figure 2B, the legend indicates NV, R, T, and S, but these notations are not found in the revised figure.**

Thanks for the comment. The figure now incorporates the gate names matching the legend (line 505).

**4. In Figure 3C, the legend indicates n=18, but the text in the results section indicates that the sample number was 9 and that each sample was quantified in duplicate.  Does the plot show the each of the duplicates.  Please clarify.**
Thanks for pointing this out. It has been corrected in the legend (line 528) and on the figure. Nine samples in duplicate as written in the text (line 331) is correct with the average plotted in the figure.


**---------------------------------------------------------------------------------------------------------------------------**

**Conclusions**

**Reviewer #1:**

**The discussion (including description of limitations, relationships between Pm and other Plasmodium species and broader significance) is significantly improved from the previous version.**

**Reviewer #2:**

**The conclusions are supported by the data presented. The discussion section nicely laid out the strengths and limitations of the study, as supported by the data and the current state of the literature.**

**---------------------------------------------------------------------------------------------------------------------------**

**Editorial and Data Presentation Modifications**

**Reviewer #1:**

**The language clarity has been significantly approved. Some additional minor comments:**

**Introduction**

**The order of sentences feels a little confusing especially given that the manuscript is focused on Pm– I would suggest the first sentence identifying Pm as important, then introduce all 5 Plasmodium species, then highlight Pf and Pv as responsible for most cases/deaths, then get to importance of Pm.**

This has been adjusted in the introduction (lines 48-53).

**Line 57 – sentence starting with “secondly” is not a full sentence. Suggest “Secondly,** **P. falciparum parasites invade and grow in RBCs of all maturities and their development ends with bursting the RBCs which can lead to severe anaemia.”**

Thanks for the suggestion. The text has been already edited (lines 58 and 59).

**Line 80 – similar issue to line 57
“Second, the reticulocyte binding protein (RBP) family that includes the *P. yoelii* 235-kDa rhoptry proteins, the *P. vivax* reticulocyte binding proteins (PvRBP-1 and -2), and the *P. falciparum* reticulocyte homology proteins (PfRH1, PfRH2a, PfRH2b, PfRH4, and PfRH5”**

This sentence has been corrected in the text (lines 81-83)

**Line 117 – “quantitative” instead of “powerful”**

Edited in line 118.

**Line 120 – add “of” after “absence”**
Edited in line 121.

**Line 285 – is this average number merozoites? Median?**

This is the average number. It has been specified in line 283.

**Figure 1B and 2C– axes are confusing. I would abbreviate “number” rather than “merozoite” (e.g. “#** **merozoites per schizont”).**
The suggestion has been incorporated in both, figures 1B (line 495) and 2C (line 505).

**Line 301 – change “that informed of the viability of the parasites” to “****to measure parasite viability”**
Edited in line 303.

**Line 312 – change “oppositely” to “****in contrast”**
Edited in line 314.

**Discussion**

**– There seems to be a lot of variability between sample response to antibodies – is it possible that some samples are responding to the antibody?**

Yes, it would be possible indeed. That is why we have detailed the limitations of the study (e.g. using a single antibody concentration) and been particularly careful in not overstating our results giving the modest sampling size.

**Line 369 – remove “of” after “despite”**
Removed in line 361.

**Line 375 – 377 – this sentence is confusing and doesn’t seem necessary**
The sentence has been removed from the text.

**Line 382-383 change to “No var genes orthologues have been identified, unlike in *P. falciparum* where PfEMP1s in the RBC membrane are encoded by var genes”.**
Edited from line 371 to 373.

**Line 418 - remove “of” after “despite”; change the “of” after “role” to “for”**
Edited in line 408.

**Line 421 – change “then “to “thus”**
Edited in line 411.

**Line 449 – change “has rendered” to “is”**
Edited in line 449.

**Line 460-461 – also mention the need to investigate samples in other geographies with different transmission patterns**
This has been incorporated in line 460.

**Reviewer #2**:

Please see above.

**--------------------**

**Summary and General Comments**

**Reviewer #1:**

**Overall, this manuscript is significantly improved. I would recommend acceptance following changes to Figure 6 (either modification or removal of figure and move of accompanying text to discussion) and have suggested some additional minor improvements. The manuscript will provide useful information to the field about the biology of *Plasmodium malariae*, an under-characterized yet important malaria parasite.**

We appreciate the positive feedback. Having adjusted figure 6, moved the corresponding paragraph to discussion and edited all the minor corrections, we think we have addressed your valuable comments.

**Reviewer #2:** (No Response).

**--------------------**

PLOS authors have the option to publish the peer review history of their article.

If published, this will include your full peer review and any attached files.
If you choose “no”, your identity will remain anonymous, but your review may still be made public.

**Reviewer #1:** Yes: Kathleen Dantzler Press

**Reviewer #2:** No


**Figure Files:**
While revising your submission, please upload your figure files to the Preflight Analysis and Conversion Engine (PACE) digital diagnostic tool, [https://pacev2.apexcovantage.com](https://pacev2.apexcovantage.com/). PACE helps ensure that figures meet PLOS requirements. To use PACE, you must first register as a user. Then, login and navigate to the UPLOAD tab, where you will find detailed instructions on how to use the tool. If you encounter any issues or have any questions when using PACE, please email us at figures@plos.org.
 
**Data Requirements:**
Please note that, as a condition of publication, PLOS' data policy requires that you make available all data used to draw the conclusions outlined in your manuscript. Data must be deposited in an appropriate repository, included within the body of the manuscript, or uploaded as supporting information. This includes all numerical values that were used to generate graphs, histograms etc.. For an example see here: [http://www.plosbiology.org/article/info%3Adoi%2F10.1371%2Fjournal.pbio.1001908#s5](http://www.plosbiology.org/article/info%3Adoi%2F10.1371%2Fjournal.pbio.1001908#s5).
 
**Reproducibility:**
To enhance the reproducibility of your results, we recommend that you deposit your laboratory protocols in protocols.io, where a protocol can be assigned its own identifier (DOI) such that it can be cited independently in the future. Additionally, PLOS ONE offers an option to publish peer-reviewed clinical study protocols. Read more information on sharing protocols at <https://plos.org/protocols?utm_medium=editorial-email&utm_source=authorletters&utm_campaign=protocols>
